# Unexpected Cardiovascular Oscillations at 0.1 Hz During Slow Speech Guided Breathing (OM Chanting) at 0.05 Hz

**DOI:** 10.3389/fphys.2022.875583

**Published:** 2022-05-10

**Authors:** Gerard Hotho, Dietrich von Bonin, Daniel Krüerke, Ursula Wolf, Dirk Cysarz

**Affiliations:** ^1^ Biologisch Onderzoek Gennep, Eindhoven, Netherland; ^2^ Research Department, Clinic Arlesheim, Arlesheim, Switzerland; ^3^ Group Practice Therapeutikum, Bern, Switzerland; ^4^ Institute of Complementary and Integrative Medicine, University of Bern, Bern, Switzerlamd; ^5^ Integrated Curriculum for Anthroposophic Medicine, Faculty of Health, Witten/Herdecke University, Witten, Germany

**Keywords:** slow breathing, heart rate variability, Mayer waves, OM-chanting, anthroposophic therapeutic speech

## Abstract

Slow breathing at 0.1 Hz (i.e., 6 cycles per minute, cpm) leads to strong cardiovascular oscillations. However, the impact of breathing below 6 cpm is rarely addressed. We investigated the influence of OM chanting, an ancient Indian mantra, with approx. 3 respiratory cpm (0.05 Hz) on the synchronisation of heart period (RR), respiration (RESP) and systolic blood pressure (SBP). Nine healthy, trained speech practitioners chanted three sequences of five subsequent OM with 2 min pauses in between. Each single OM chanting consisted of taking a deep breath and a long “OM” during expiration and lasted approx. 20 s. ECG, respiration and blood pressure were recorded continuously, of which the RR tachogram, RESP and SBP were derived. Synchronisation between the signals was computed using the phase difference between two signals. During OM chanting synchronisation among the oscillations of RR, SBP and RESP was significantly increased compared to rest. Furthermore, OM chanting at breathing frequencies between 0.046 and 0.057 Hz resulted in 0.1 Hz oscillations in RR and SBP. In conclusion, OM chanting strongly synchronized cardiorespiratory and blood pressure oscillations. Unexpected oscillations at 0.1 Hz in SBP and RR appear at breathing frequencies of approx. 0.05 Hz. Such frequency doubling may originate from an interaction of breathing frequency with endogenous Mayer waves.

## Introduction

Influences of different breathing frequencies and patterns on cardiovascular regulation have been investigated in the past decades (Hirsch and Bishop, 1981; Berntson et al., 1993; Laude et al., 1993; Klintworth et al., 2012). It has been shown that slow breathing especially at slow rates (e.g. 6 cycles per minute) leads to enhanced cardiovascular oscillations at the respective frequency. Promising results have been attained regarding the effectiveness of biofeedback inducing slow deep breathing in treating depression, anxiety, asthma, fibromyalgia, posttraumatic stress disorder and other conditions (Szulczewski and Rynkiewicz, 2018). Chanting of mantras or other religious practices also decrease the breathing frequency and, hence, lead to similar effects like biofeedback (Bernardi et al., 2001) and as a long-term effect, cardiovascular functioning may be enhanced (Mooventhan and Khode, 2014). In contrast to device guided breathing, the chanting of mantras adds an aesthetic and spiritual dimension to the physiological effects. These dimensions may have an impact on the state of mind and this may even be essential for the efficacy of slow breathing exercises, e.g. in the treatment of hypertension (Dick et al., 2014). One prominent example of mantras is OM. In ancient Indian scriptures, OM is the symbol of God Brahman (Kumar et al., 2010) and OM covers the whole threefold experience of man: It is the “shortened” combination of three letters, namely, A, U, and M. “A” represents the physical plane and “U” represents the mental and emotional plane, “M” represents the deep-sleep state, which is unknown to wakeful consciousness (Kumar et al., 2010).

During meditative chanting of OM, a state of mental alertness is induced (Kumar et al., 2010), which is accompanied by physiological effects such as the coordination of blood pressure oscillations (Mayer waves), cerebral blood flow and heart rate variability, HRV (Bernardi et al., 2001). At the same time, an increase in arterial baroreflex sensitivity has been observed (Bernardi et al., 2001). After loud OM-chanting, a significant increase in theta power was found in the EEG when averaged across all brain regions, indicating a lasting effect of relaxation (Harne and Hiwale, 2018).

Functional magnetic resonance imaging in healthy individuals showed reduced outputs from the insula, anterior cingulate and orbitofrontal cortices during OM chanting (Kalyani et al., 2011; Rao et al., 2018). Of possible therapeutic value is the observed reduction of outputs from these regions to the amygdala, since a structural hyperconnectivity involving the amygdaloid nuclei in the right hemisphere occurs in major depressive disorder (Brown et al., 2019). This may indicate that OM chanting activates neural structures involved in attention, emotions and control of the autonomic nervous system. There are also indications that Pranayam respiratory training modulates ventricular performance by increasing parasympathetic activity and decreasing sympathetic activity (Udupa et al., 2003).

It has been shown that oscillations of different physiological systems such as heart period and respiration may interact and intermittently synchronise (Schäfer et al., 1998; Bartsch et al., 2012; Bashan et al., 2012) and it is known that slow breathing (e.g. 6 cycles per minute) in the context of mantra-recitation leads to strong cardiovascular oscillations (Bernardi et al., 2001). However, the impact of even slower breathing at e.g., 3 cycles per minute is rarely addressed. Zen, Tanden breathing at 3 to 4 cycles per minute increased the oxygenated haemoglobin level and the alpha band activity in the electroencephalogram (EEG) whereas the theta band activity in the EEG decreased compared to spontaneous breathing (Zaccaro et al., 2018). Furthermore, after expiratory breath hold a single inspiration leads to one full oscillation in heart period and blood pressure (Penaz and Burianek, 1963) similar to what would be expected from one complete respiratory cycle mediated via respiratory sinus arrhythmia.

OM chanting induces slow breathing at approximately 3 cycles per minute. This breathing rate is considerably slower than breathing at 6 cycles per minute which is often associated to “slow breathing”. It leads to a strong coordination and interaction of cardiovascular oscillations with respiration, involving central and peripheral neural networks (Telles et al., 1995), thus suggesting a state of coherence between physiological and mental functions.

These effects are not limited to the intervention itself. The increase in cardiorespiratory coordination persists after slow breathing for 15–20 min and similarly after recitation of ancient verse (Bettermann et al., 2002; Dick et al., 2014). Such recitation is applied regularly in Anthroposophic Therapeutic Speech (ATS), which also utilizes rhythmic language for the treatment of cardiovascular disease (Cysarz et al., 2004; Kruerke et al., 2018). Slow breathing of 15 min for 30 days also enhanced subjective sleep quality and cardiac vagal activity (Laborde et al., 2019). Voicing of hexameter (standard epic metre in classical Greek literature, e.g., Iliad, Odyssey) induces a higher synchronisation of heart period and respiration than paced breathing at the same frequency (Cysarz et al., 2004). Hence, both recitation of hexameter and OM chanting have become useful therapeutic tools.

In a preliminary study performed by our research group, we observed that two heart period oscillations appeared during one OM breathing cycle (von Bonin et al., 2001). Interestingly, only one out of the two oscillations could be attributed to the OM chanting induced respiration frequency of 0.05 Hz, whereas the second oscillation remained of unknown origin. For this reason, the aims of the current study were 1) to further elucidate how respiration, blood pressure and heart period oscillations interact during OM chanting and 2) to characterize the appearance of these non-respiratory related oscillations. The first aim is investigated analysing the synchronization among the different signals. The second aim is elucidated by analysing the appearance of the second oscillation in relationship to the breathing frequency of the OM chant.

## Methods

### Experimental Design

After providing written informed consent, subjects were enrolled in the study. All subjects were healthy and professional speech and drama therapy practitioners. The practitioners had a personal interest in understanding the physiological mode of action underlying ATS and keen to aid in this research. The study protocol was approved by the Ethics Committee of the Canton Zurich (KEK-ZH-Nr. E-50/2002).

From originally ten subjects, one had to be excluded due to frequent ectopic heartbeats. The remaining nine subjects were included and had no history of cardiovascular diseases, especially no hypo- or hypertension. The nine subjects consisted of five female and four male volunteers, age 41 ± 7.6 years, BMI 22.8 ± 3.2 kg/m^2^. All subjects were familiar with OM chanting.

Each subject took part in one experimental session of 30 min. The session consisted of a pre-resting period of 10 min (baseline), three sequences of five subsequent OM chantings with a 2 min pause between the sequences and a post-resting period of 10 min (recovery). All phases of the experiment were performed in standing position. During pauses between the OM chanting and during the resting periods the subjects breathed spontaneously. OM chanting was instructed to be full-toned at normal pitch and on one breath and the duration of each OM to be approximately of the same length within an individual subject. No pacing was introduced to allow for a natural interindividual variance in chanting pace.

During each session, the ECG (lead II) with automatic R-peak detection (sampling rate 128 Hz for ECG and 4,096 Hz for R-peaks) and the nasal/oral airflow (sampling rate 64 Hz) were recorded by a Holter device (MK-3 Medikorder, Tom Medical, Graz, Austria). The uncalibrated continuous blood pressure using a finger-cuff measurement device (Portapres; Finapres Medical Systems, Amsterdam, Netherlands, sampling rate 100 Hz) was simultaneously continuously recorded during the entire session.

### Data Analysis

The automatically detected R peaks were visually examined using both the ECG and the RR tachogram and manually corrected for ectopic beats. The beat-to-beat systolic blood pressure and a blood pressure based interbeat interval series (corresponding to the RR tachogram) were derived from the continuous blood pressure signal using dedicated software. The blood pressure derived interbeat interval series was visually inspected and manually corrected for outliers as well. Subsequently, ECG and respiration were time matched with the continuous blood pressure signal by matching their RR tachograms, after both had been resampled to 10 Hz. Respiratory cycles were derived from the nasal/oral airflow using successive local minima.

### Baroreflex-Sensitivity

BRS was computed according to the sequence method (Bertinieri et al., 1988; Di Rienzo et al., 2001), hence x_SBP_ (n-1) was paired with x_RR_ (n), with x_SBP_ (n) and x_RR_ (n) being the nth sample of the systolic blood pressure beat-to-beat series and RR tachogram, respectively. Furthermore, the minimal number of elements in each BRS sequence amounted to 3, the minimal absolute difference in SBP between two consecutive samples was 1 mmHg and the minimal absolute difference in RR equalled 5 ms between two consecutive samples. Moreover, the minimal coherence in each sequence had to be at least 0.8. It was verified that a sufficient number of data elements were available for computing the BRS (between 15–121 for OM and the resting periods), so that the abovementioned method led to reliable statistical results (Gouveia et al., 2009). One median BRS value was computed for each subject during OM chanting (BRS_OM_), pre-resting (BRS_PRE_) and post-resting (BRS_POST_).

### Synchronisation

Synchronisation between respiration, RR tachogram and beat-to-beat systolic blood pressure was analysed using the oscillations in heart period as well as in systolic blood pressure induced by respiration (respiratory sinus arrhythmia, RSA). To determine synchronisation between two signals, the cross-correlation function may be used. However, a parameter of synchronization γ has been proposed to better discriminate synchronisation during varying activity levels (Cysarz et al., 2004). This parameter denotes the amount of synchronisation between two signals, 0 ≤ γ ≤ 1, where signals that are fully synchronized in a statistical sense have a value of γ = 1 and signals that are completely desynchronized have a value of γ = 0. Without going into detail, this parameter was calculated by first performing the Hilbert transform of each of the two band-pass filtered signals, resulting in their phase signals as a function of time. Thereupon, the difference of these two-phase signals was analysed for variation over time (no variation implies full synchronisation), where phase jumps of 2π had to be corrected for. The measure of this variation over time is γ. A detailed description of this method is found in (Cysarz et al., 2004).

**FIGURE 1:**
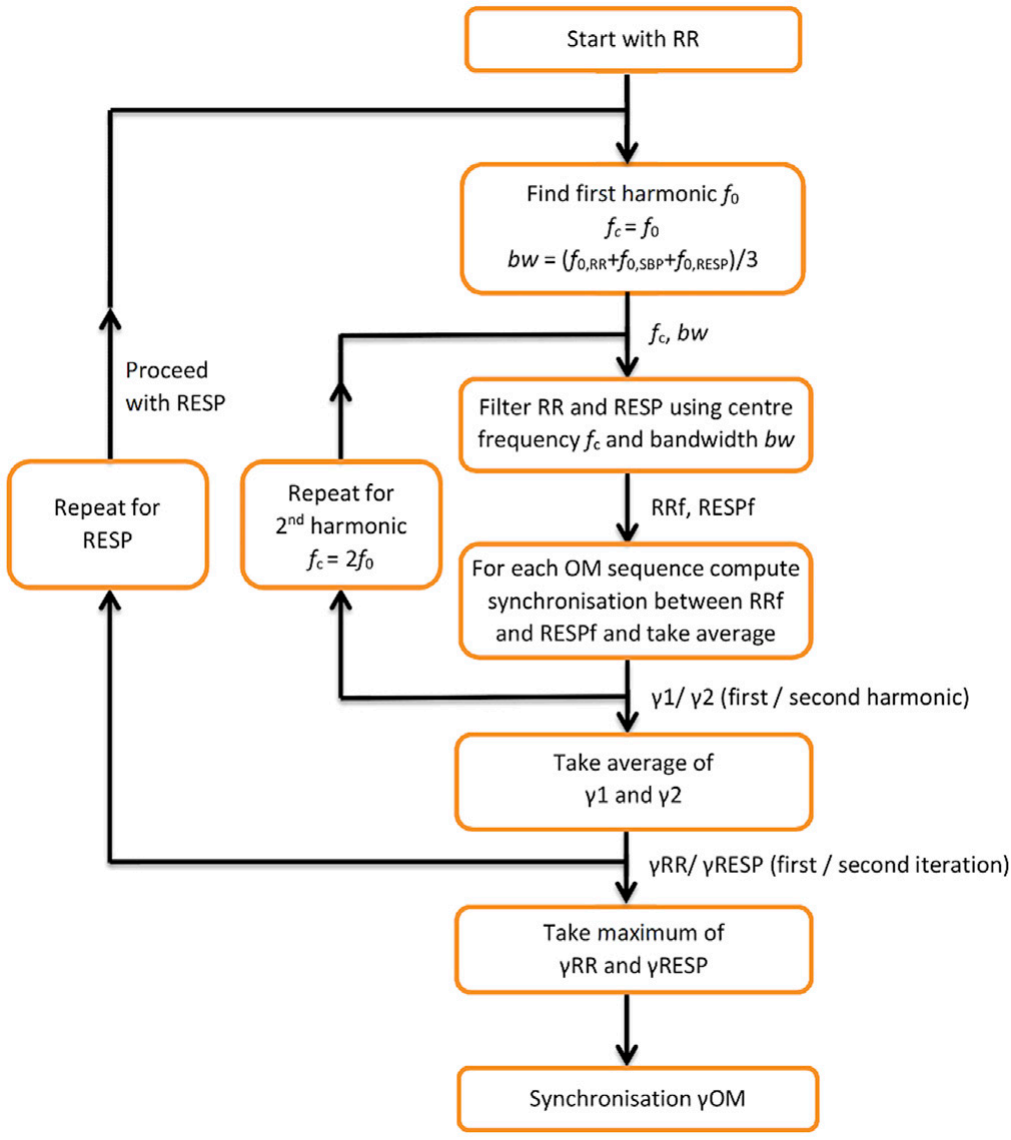
Flow diagram for computing synchronisation between RESP and RR during OM. f_0_—first harmonic; f_c_—centre frequency; bw—bandwidth; RR_f_, RESP_f_—band passed RR tachogram and RESP signal at centre frequency f; y_1_, y_2_—synchronisation indices for the first and second harmonic; γ_RR_—average of y_1_, y_2_; γ_RESP_—synchronisation index applied to RESP as a reference; γ_OM_—resulting synchronisation index.

A flow diagram for computing synchronisation between RR and RESP during OM is depicted in Figure 1. It starts with finding the first harmonic of RR, f_0,RR_, and applying a band-pass filter with centre frequency f_c_ = f_0, RR_ and bandwidth bw = (f_0, RR_ + f_0, SBP_ + f_0, RESP_)/3 to both RR and RESP. Next, for each of the 3 OM sequences synchronisation between the filtered RR and RESP signals is determined and its average value equals γ_1_. For the second harmonic of RR, the same procedure is repeated, except that f_c_ = 2f_0, RR_. This results in synchronisation γ_2_. The average of γ_1_ and γ_2_ is γ_RR_. Subsequently, the same procedure as for RR is applied to RESP as a reference, resulting in synchronisation γ_RESP_. Synchronisation γ_OM_ between RR and RESP is taken as the maximum value of γ_RR_ and γ_RESP_.

For computing synchronisation between RR and RESP during pre- and post-resting periods, we applied a sliding window of length T_OM_, with 50% overlap to RR and RESP, where T_OM_ is the sum of the time lengths of the 3 OM sequences. To each pair of corresponding RR and RESP data window, the same procedure as during OM was applied for computing synchronisation, with two exceptions. First, we created 3 separate sequences by splitting the data in the sliding window in 3 non-overlapping windows of equal length, to proceed identically as with the 3 OM sequences. Secondly, because RR and RESP hardly showed harmonics during the resting periods, we took for the first spectral peak the largest in the frequency range between 0.0375 and 0.2 Hz (the first spectral peak during OM was located between 0.046 and 0.095 Hz). This resulted in synchronisations γ_PRE_ and γ_POST_.

Using the method described above, one value per subject for γ_OM_ was obtained and a set of values for γ_PRE_ and γ_POST_ (depending on the length of the sliding data window 3–6 values each).

The combination RR and SBP and the combination RESP and SBP were analysed analogously. We therefore obtained for each subject: 3 values for γ_OM_ (1 value for RR-RESP, RR-SBP and RESP-SBP each), between 9 and 18 values for γ_PRE_ (3–6 values for RR-RESP, RR-SBP and RESP-SBP each) and between 9 and 18 values for γ_POST_.

### Statistical Analysis

All statistical analysis was performed using non-parametric statistical procedures. Hence, median values and the interquartile range (IQR) were used to quantify the distributions. Differences in the RR interval, the respiratory cycle (RESP), BRS and synchronisation γ during pre-resting, OM and post-resting periods were analysed by the Friedman test. In case of significant difference, the Wilcoxon signed rank test was used to examine which of the three pairs (OM—pre-resting, OM—post-resting and pre-resting—post-resting) were significantly different, using the Bonferroni correction for multiple testing.

Since uncalibrated SBP values were recorded, i.e., each blood pressure measurement (subject) had a different offset, we performed a Wilcoxon signed rank test to SBP differences: we calculated for each subject ΔSBP_OM-PRE_ = SBP_OM_—SBP_PRE_, ΔSBP_OM-POST_ = SBP_OM_—SBP_POST_ and ΔSBP_PRE-POST_ = SBP_PRE_—SBP_POST_, where SBP_PRE_, SBP_OM_ and SBP_POST_ are the median systolic blood pressure during pre-resting OM and post-resting periods, respectively. To correct for multiple testing the Bonferroni correction was applied. A p-value < 0.05 was considered statistically significant for all tests.

## Results

**FIGURE 2:**
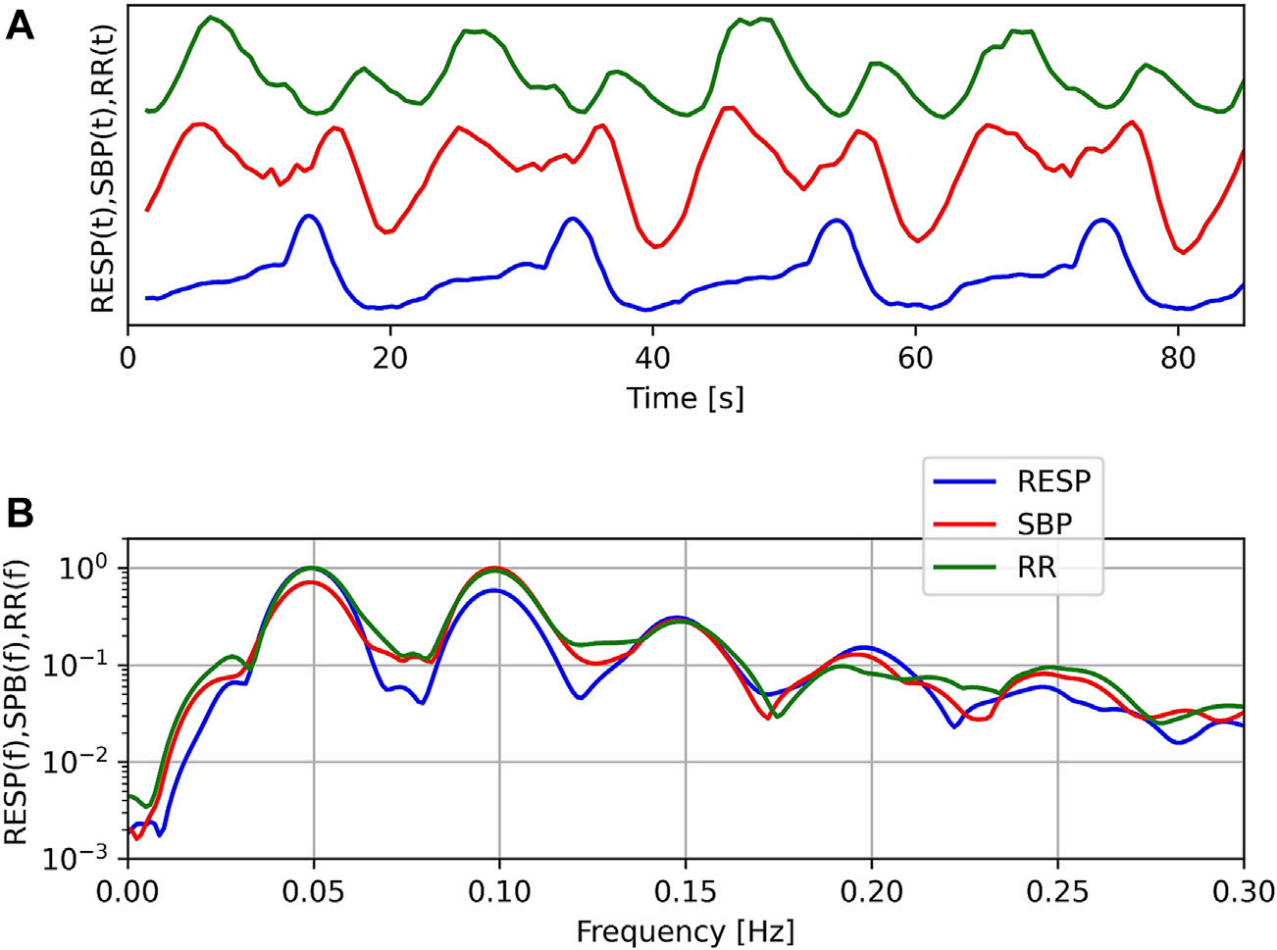
Example of respiration (RESP), systolic blood pressure (SBP) and RR-tachogram (RR) of one subject during OM chanting **(A)** and corresponding normalised spectra **(B)**. Note: In the top figure, no units are given, since we compare the temporal structure of the signals in time and the offset of SBP is unknown.

The top panel of Figure 2 shows a typical example of the RR tachogram (RR), systolic blood pressure variations (SBP) and respiration (RESP) during OM chanting in one subject. Four OM chants can easily be discerned from the RESP signal. Obviously, RR and SBP show two oscillations during each OM chant. Especially the RR tachogram shows oscillations with alternating amplitude. The inspiration as indicated by the local maximum to the following local minimum in RESP leads to an oscillation with lower amplitude and OM, i.e. expiration, leads to an oscillation with a larger amplitude. The bottom panel shows the normalised spectra of these signals. Normalisation was obtained by dividing each spectrum by its maximum value. The major frequency is approx. 0.05 Hz corresponding to a respiratory frequency of three cycles per minute. All spectra include harmonics, at least 3 per signal.

### Respiratory Cycle, RR Interval, Systolic Blood Pressure

**FIGURE 3:**
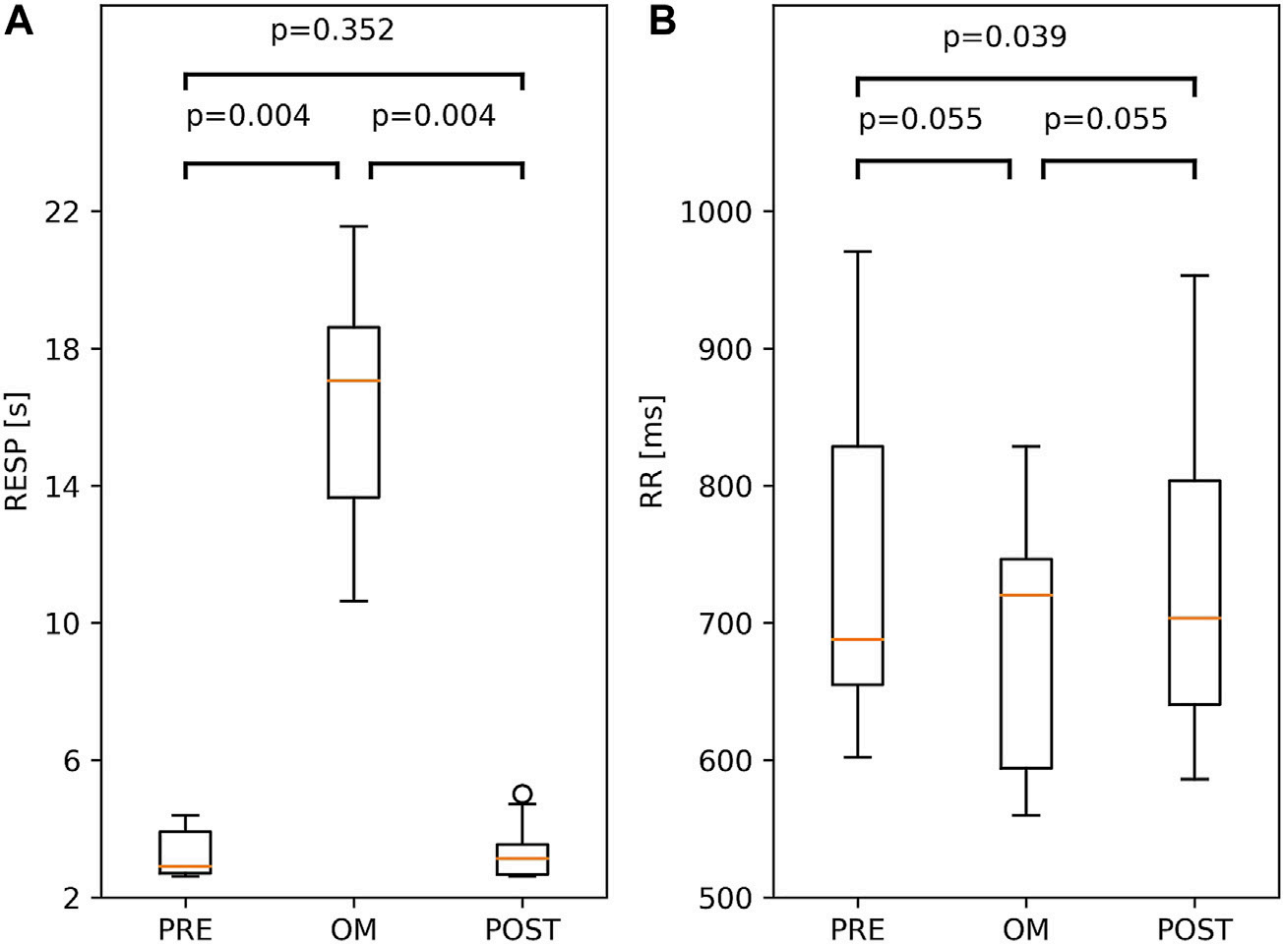
Boxplots of Respiratory period, RESP, **(A)** and RR interval; RR, **(B)** during OM, pre-resting and post-resting. The probability of similar values between two conditions is indicated by the p-values above the box and whisker plot. The boxplots show median and quartiles (horizontal lines), and maximum and minimum values (whiskers).

Both for the respiratory cycle and the RR interval, the Friedman test showed considerable variation across pre-resting, OM and post-resting (p = 0.001 and p < 0.01, respectively). In the left panel of Figure 3, the respiratory cycle during pre-resting, OM and post-resting is shown; a large and significant difference was found between OM and both resting periods (>14.0 s), but not between both resting periods. For the RR interval, which is shown in the right panel of Figure 3, no significant differences were found among the three pairs after the Bonferroni correction.

**FIGURE 4:**
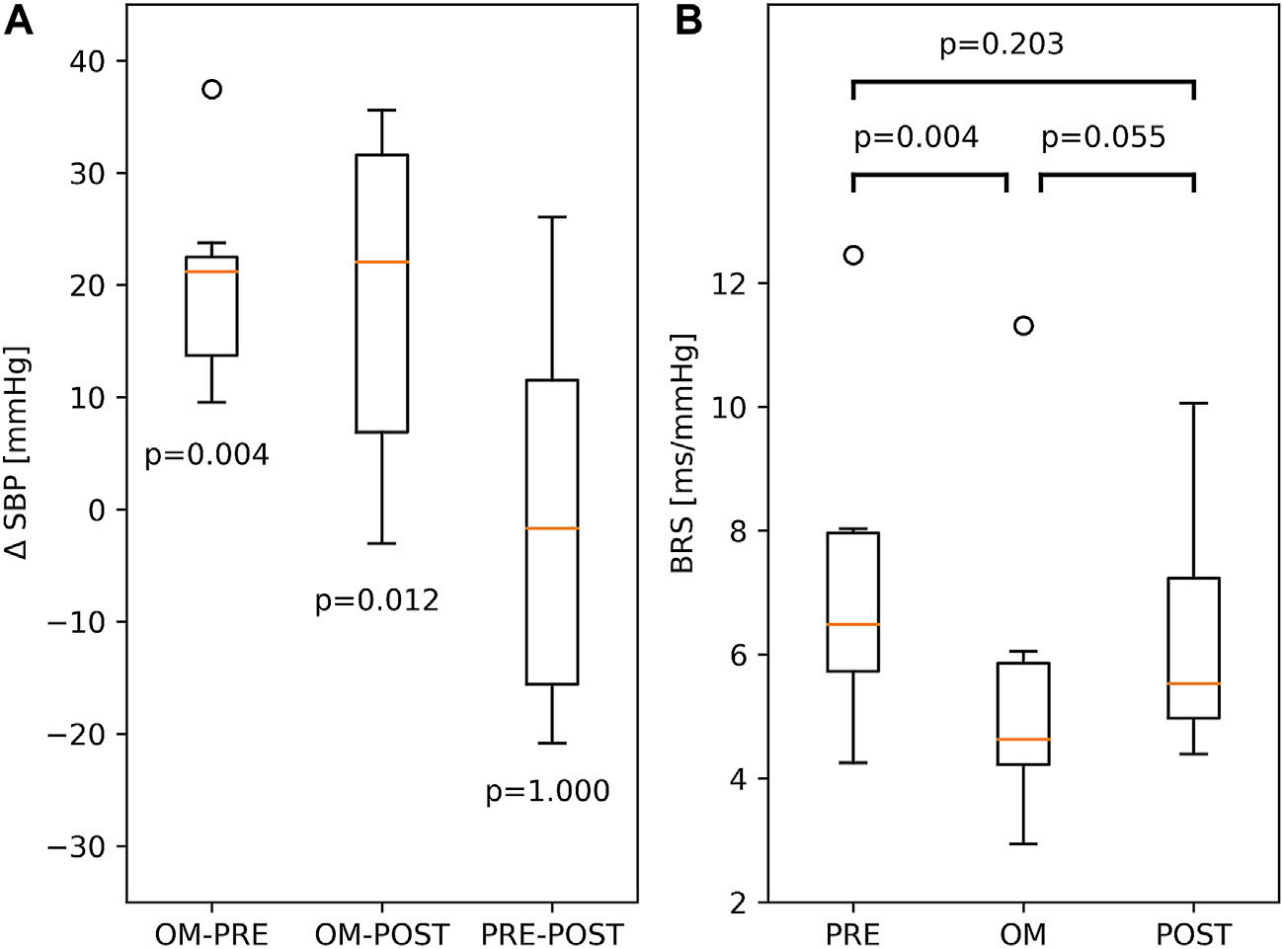
**(A)** Boxplots of differences between SBP, ΔSBP, in PRE-resting, OM and POST-resting condition. The p-values indicate the probability of ΔSBP to be different to zero. **(B)** BRS during PRE-resting, OM and POST-resting condition. The probability of similar values between two conditions is indicated by the p-values above the box and whisker plot. The boxplots show median and quartiles (horizontal lines), and maximum and minimum values (whiskers).

Since SBP was not calibrated, we investigated differences in SBP. The difference in SBP between OM and pre-resting for a particular subject is ΔSBP = SBP_OM_—SBP_PRE_. The differences in SBP between OM and post-resting, and between pre-resting and post-resting are defined analogously. In the left panel of Figure 4, all three ΔSBP across all subjects are depicted. A distinct and significant difference in SBP was found between OM and both resting periods (>21.0 mmHg). In the two resting periods SBP was similar.

### Baroreflex-Sensitivity

BRS showed significant variation across pre-resting, OM and post-resting (p < 0.01). BRS during pre-resting was significantly higher than during OM. Between OM and post-resting and between pre- and post-resting, no significant differences were found (right panel of Figure 4).

### Synchronisation

**FIGURE 5:**
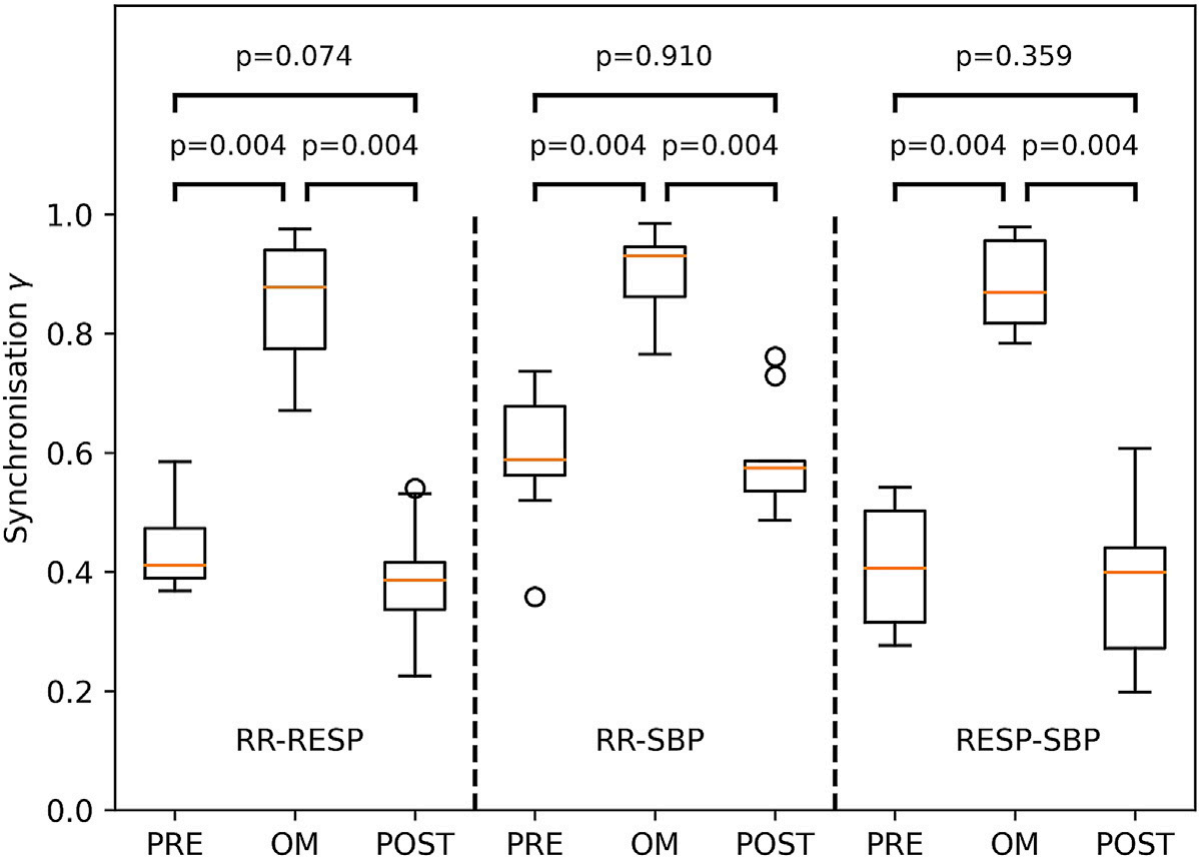
Boxplots of the synchronisation index γ between RESP and RR (left part), RR and SBP (middle part) as well as RESP and SBP (right part) during PRE-resting, OM and POST-resting condition. The probability of similar values between two conditions is indicated by the p-values above the box and whisker plot. The boxplots show median and quartiles (horizontal lines), and maximum and minimum values (whiskers).

The Friedman test showed considerable variation across pre-resting, OM and post-resting for synchronisation γ of RR-RESP (p < 0.001), RR-SBP (p < 0.01) and RESP-SBP (p < 0.001). Synchronisation in all subjects during OM chanting and both resting periods is shown in Figure 5. Synchronisation during OM was always significantly stronger than during both pre-resting and post-resting periods (median values for pre-resting, OM and post-resting during RR-RESP: 0.41, 0.88, 0.39; RR-SBP: 0.59, 0.93, 0.57; RESP-SBP: 0.41, 0.87, 0.40). Synchronisation during pre-resting and post-resting was similar, which indicates that there was no carry-over effect of OM chanting to the post-resting period with respect to synchronisation between signals.

The time order of the signals was always RESP—SBP—RR. The median (IQR) phase differences amounted to ɸ_RESP-SBP_ = 0.26π (0.18π to 0.46 π) and ɸ_RESP-RR_ = 0.52π (0.38π to 0.59 π), which corresponded with a delay of 2.33 (1.47–3.27) seconds for SBP and of 3.84 (3.33–4.80) seconds for RR intervals.

### Double Oscillations in Beat-To-Beat SBP and RR Tachogram During OM

**FIGURE 6:**
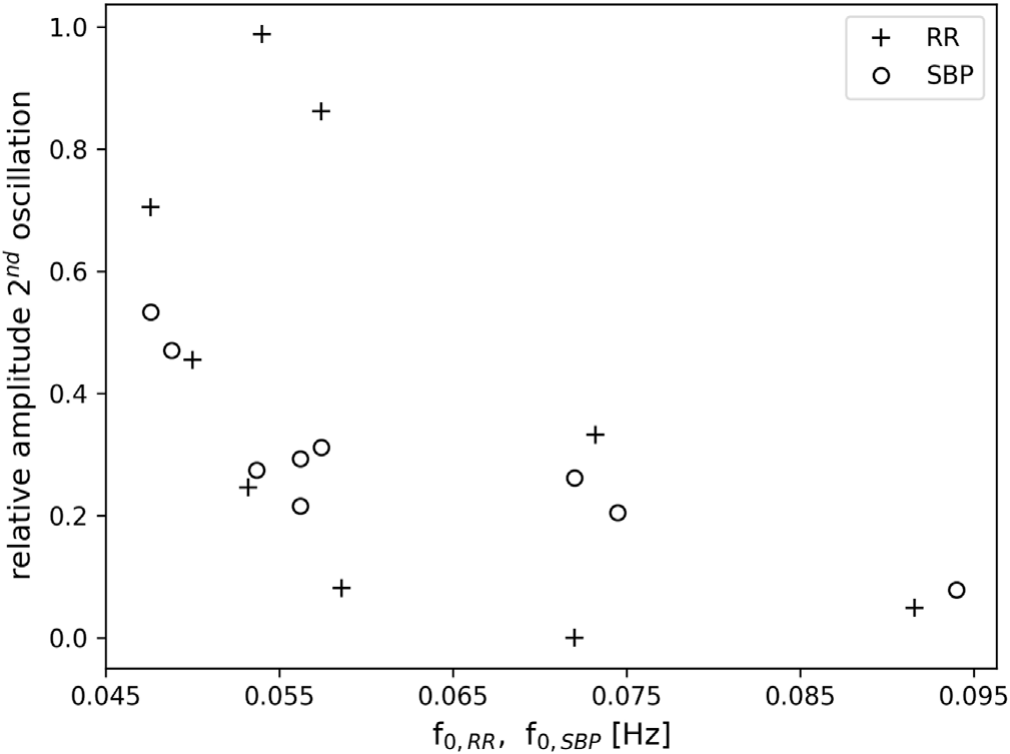
Relative amplitude of a second oscillation in the RR tachogram (+) and SBP (o) as a function of the frequency of the first harmonic f_0,RR_ and f_0,SBP_, respectively. Note, that the first harmonic in RR and SBP corresponds to the breathing frequency.

During one OM chant, a clearly visible non-respiratory second oscillation appeared in the time signals of SBP and RR intervals (c.f. example in Figure 2), which seemed to depend on the breathing frequency. To quantify the amplitude of the second oscillation, its amplitude was calculated relative to the amplitude of its preceding oscillation. A relative amplitude close to 1 implies that the amplitude of the second oscillation was as high as its preceding oscillation. If the relative amplitude is close to 0 then the second oscillation is not visible, i.e. a second oscillation does not appear. Figure 6 shows the average relative amplitude of the second oscillation in the RR tachogram and SBP as a function of the first harmonic of the RR intervals, f_0,RR_, and the first harmonic of the SBP, f_0,SBP_, respectively (the first harmonics of the RR intervals, SBP and RESP were very similar during OM). A second oscillation with an amplitude almost as high as in the preceding oscillation (relative amplitude >0.7) appeared in the RR tachogram of three subjects. These oscillations occurred at frequencies of the first harmonics in the range 0.046–0.057 Hz. There were also three subjects that hardly showed a second oscillation in the RR tachogram. They showed relative amplitudes <0.1 and had frequencies of the first harmonic between 0.057 and 0.092 Hz. Furthermore, three subjects showed intermediate levels of the amplitude of the second oscillation (frequency range 0.05–0.073 Hz). For the SBP time series the amplitude of the second oscillation was in the intermediate range (relative amplitude >0.2 and <0.6, frequency range 0.048–0.075 Hz) for all subjects except one (at 0.094 Hz).

## Discussion

In this explorative study on the effects of OM chanting in healthy professional speech and drama therapy practitioners the subjects performed the experiment in standing position and rested before, after and between the OM chanting, breathing spontaneously. During OM, the breathing rate dropped from an average 19.9 cycles per minute to 3.5 cycles per minute, due to the intervention whereas the RR-interval did not change significantly. Systolic blood pressure increased by > 21.0 mmHg during OM-chanting.

In the present study the OM chanting was carried out in standing posture because respiratory variables like e.g. the vital capacity can be maximized in this posture (Lalloo et al., 1991; Price et al., 2014) which is essential for respiratory frequencies below 0.1 Hz. However, previous studies on slow paced breathing often used a supine or sitting position, i.e. conditions without orthostatic stress. It has been shown that the body position affects cardiac autonomic regulation and cardiorespiratory regulation during slow paced breathing at 0.1 Hz. Complexities on short and long-term scales and also self-similarities of the RR interval series change between standing and supine position as well as between spontaneous breathing and slow-paced breathing at 0.1 Hz (Matic et al., 2020). Furthermore, the coherence between interbeat intervals and respiration was lower during standing compared to the supine position. Hence, the effects of OM chanting on synchronization between respiration and the RR interval series may be attenuated by the standing posture.

We did not find an increase in BRS during OM-chanting induced by the slow breathing as described in previous studies (Pitzalis et al., 1998; Horsman et al., 2015). These authors demonstrated a dependence of baroreflex control on breathing rate, using respiratory frequencies of 6–16 cycles per minute and finding an increase in baroreflex gain during slower breathing in normo- and hypertensive subjects (Joseph et al., 2005). The OM chanting in our experiment induced very low respiratory frequencies of 3–6 cycles per minute and lead to a decrease in BRS. This decrease may partially be attributed to the lower respiratory frequencies and also to the difference in posture: the previous studies were carried out with subjects in supine or sitting position, i.e. without orthostatic stress and, hence, with lower sympathetic activity (Cooke et al., 1999) leading to a more pronounced BRS.

Respiration at low frequencies below 0.1 Hz (i.e. below 6 cycles per minute) has been investigated sparsely. To the best of our knowledge only one study investigated respiratory frequencies in the range of 0.01–0.143 Hz with respect to its impact on heart period and SBP (Vaschillo et al., 2002). In this study, increases in respiratory frequency from 0.01 to 0.143 Hz lead to an augmentation of oscillations in heart periods. The strongest influence of respiration on heart period oscillations was observed at approx. 0.1 Hz. The heart period oscillations slightly preceded respiration for respiratory frequencies close to 0.05 Hz (phase difference approx. −0.1π), whereas they were slightly delayed for frequencies close to 0.1 Hz (phase difference approx. 0.1π). At the same time, at a respiratory frequency of 0.05 Hz, SBP variations were delayed by approx. 0.1π with respect to respiration.

During OM chanting the median delay of SBP and RR interval series with respect to respiration was 0.52π and 0.26π. These results differ slightly from Vaschillo’s findings (Vaschillo et al., 2002). However, it should be noted that OM chanting was carried out with a shorter inspiration and long OM chanting, i.e., a long expiration (inspiration to expiration ratio <1). In contrast, the preliminary study used sinusoidal respiration for all respiratory frequencies (inspiration to expiration ratio close to 1). The inspiration to expiration ratio may have an impact on the delay between the signals. Nevertheless, the present results are in qualitative agreement with the previous findings.

The subjects in our experiment did not show signs of hyperventilation, which decreases BRS (Bourdillon et al., 2018). However, the prolonged exhalation during OM chanting may have reduced the arterial carbon dioxide partial pressure, influencing the CO_2_ sensitive central and peripheral chemoreceptors and thus contributing to the observed reduction in BRS. This effect may be enhanced by the conscious control of breathing in our experiment (Bristow et al., 1974).

We observed a high degree of synchronisation among the rhythms of respiration, systolic blood pressure and heart period, as expressed by RR intervals, during OM chanting. Synchronisation was significantly lower during pre- and post-resting. Such synchronisation during slow breathing (Dick et al., 2014), hexameter recitation (Cysarz et al., 2004) and OM chanting (Bernardi et al., 2001) has already been observed.

The synchronisation of cardiorespiratory oscillations was not maintained in the post-resting period in most subjects. The absence of an after-effect might be due to the one-time intervention, which is in accordance with our findings in a single intervention of hexameter recitation, paced breathing and spontaneous breathing where a strong synchronisation of respiration and HRV during intervention appeared, but no immediate after-effect (Cysarz et al., 2004). After some weeks of training, a persisting effect of slow breathing (Dick et al., 2014; Laborde et al., 2019; Szulczewski, 2019) and hexameter recitation (Bettermann et al., 2002) on cardiovascular regulation has been demonstrated. We assume that a prolonged training in OM chanting would yield a similar effect.

### Non-Respiratory Related Oscillations

As in our preliminary study (von Bonin et al., 2001), a second oscillation appeared in the time signals of RR intervals and SBP during one OM chant, i.e., one respiratory-related oscillation, which was initiated by the inspiration (Penaz and Burianek, 1963), and another oscillation that obviously appeared without any trigger. If the breathing frequency elicited during OM chanting is at 0.05 Hz (i.e., 3 cycles per minute) the second oscillation in SBP and RR intervals is most distinct, indicating an oscillation at 0.1 Hz (Figure 6).

Which mechanisms have been reported to explain the behaviour of the RR and SBP oscillations observed? Slow breathing at 0.1 Hz in a resting condition (sitting or supine) leads to a “baroreflex resonance” called Traube-Hering waves (Vaschillo et al., 2002; Vaschillo et al., 2006; Billman, 2011; Sevoz-Couche and Laborde, 2022). I.e. a slow inspiration decreasing heart period will cause a surge in blood pressure due to the increasing amount of circulating blood. This response shows a time delay of approx. 5 seconds, caused by the inertia of the blood flow through the vascular system (Sevoz-Couche and Laborde, 2022). Hence, at a respiratory frequency of 0.1 Hz, a complete cycle of Traube Hering wave lasts approx. 10 s. As described, both oscillations are out of phase (phase difference approx. π). This baroreflex resonance is vagally mediated (Kromenacker et al., 2018; Sevoz-Couche and Laborde, 2022) and seems to involve pulse pressure modulations as well (Barnett et al., 2020).

Besides the Traube Hering waves, the so-called Mayer waves occur in the form of blood pressure oscillations at 0.1 Hz independent of respiration (Julien, 2006; Julien, 2020). Function and origin of the Mayer waves, with a central tendency at 0.1 Hz in humans, appearing in sympathetic nerve discharge, blood-pressure and HRV are still under debate. They may consist of two components, one originating in the baroreflex loop and the other in a centrally generated oscillation (Julien, 2006). The latter may be located in the pons/brain stem and drive cardiac autonomic oscillations with a short delay (Pfurtscheller et al., 2020). There is also evidence for a capacity of the spinal cord to generate such oscillations independent of supraspinal inputs (Inoue et al., 1991; Montano et al., 2000), which would lead to the assumption of a spinal source for Mayer waves (Koh et al., 1994). To complete these observations, an independent oscillation in the 0.15 Hz rhythm band in cutaneous blood flow has been described (Perlitz et al., 2004a; Perlitz et al., 2004b) and confirmed by recordings of similar blood-oxygen level-dependent oscillations (Pfurtscheller et al., 2019). If respiration rate came close to 0.15-Hz, a strong synchronisation of cardiovascular and respiratory oscillations occurred. However, simultaneous cardiovascular and respiratory oscillations at about 0.1 Hz did not affect the appearance of the 0.15-Hz rhythm in blood flow (Perlitz et al., 2004a; Perlitz et al., 2004b). Taken together, there is accumulating evidence for a neurogenic origin of cardiovascular oscillations at approx. 0.1 Hz as well as support for the hypothesis of the cardiovascular system generating these oscillations (Blinowska et al., 2020).

In the present study, Traube Hering waves are unlikely to generate the observed second non-respiratory related oscillation. The inspiration during OM chanting could have initiated Traube Hering waves. However, respiration and oscillations in heart period during OM chanting are not out of phase by π as they should be in the case of Traube Hering waves (Sevoz-Couche and Laborde, 2022). Instead, the standing position during OM chanting will elicit orthostatic stress and, hence increase sympathetic activity (Cooke et al., 1999). This will result in increasing blood pressure activity during OM chanting and may enhance the appearance of Mayer waves.

However, the present data cannot answer this question unambiguously. Previous results indicate that the cardiovascular response on respiration at 0.05 Hz is different to respiration at 0.1 Hz (Vaschillo et al., 2002). Hence, studies investigating respiration at 0.05 Hz with different inspiration to expiration ratios would be needed to further clarify the origin of the second non-respiratory related oscillation observed.

The non-respiratory oscillations at 0.1 Hz in SBP and RR interval in our experiment occur predominantly at a breathing frequency half of the Mayer waves (1:2 ratio). At slightly higher respiration frequencies (close to but still below 0.1 Hz) the second non-respiratory oscillation attenuates. Hence, the second non-respiratory oscillation does not originate from entrainment with e.g. blood pressure oscillations. Such an entrainment would lead to enhanced oscillations instead of diminished oscillations. Instead, it seems that the second oscillation only appears if there is enough time for a complete oscillation. It has been suggested that the human organism tends to establish a coordinated time structure of physiological functions such as heart period and respiration (Hildebrandt, 1986; Hildebrandt, 1991). This time structure is reflected by e.g. whole number frequency coordination of their oscillations. This endogenous time structure prefers integer number relationships in the spontaneous rhythms (Hildebrandt, 1986; Hildebrandt, 1991). In line with this assumption, most of our study subjects preferred a breathing frequency for OM chanting with an integer number ratio (1:2) to the Mayer waves (e.g. the study subject in Figure 2). Where this ratio was not maintained (faster breathing), the second oscillation disappeared. We do not expect confounding effects of varying stress levels since all speech practitioners were experienced and deliberately chose the individual frequency of OM chanting.

The emergence of Mayer waves may also have contributed to the higher statistical dependence between RR and SBP compared to RESP and both SBP and RR during resting: RESP is largely independent of both SBP and RR, whereas SBP and RR are much more correlated. Moreover, we often observed a main frequency component of SBP and RR of approximately 0.1 Hz which explains the larger value for both γ_PRE_ and γ_POST_ in RR-SBP as compared to RR-RESP and RESP-SBP (Figure 5).

The time order of the oscillations is always RESP-SBP-RR if the second oscillation in SBP and RR (Figure 1) is used to establish this time order, i.e., the single oscillation in respiration always precedes the second oscillation in both SBP and RR. Physiologically, the speech induced respiration triggers an increase in blood pressure and heart period oscillations (respiratory sinus arrhythmia) which, in the case of slow breathing at approximately 0.05 Hz, is complemented by a second oscillation of SBP and RR of approximately 0.1 Hz. In the present study, we were able to demonstrate this effect but not to establish its origin.

### Limitations

The subjects showed slightly varying patterns of OM chanting leading to partially different responses on cardiovascular regulation. Future studies should use a stricter OM chanting pattern to reduce the variance in the responses on the cardiovascular regulation. Furthermore, the possibility of gender differences in cardiovascular responses (Voss et al., 2015) should be considered in future studies. The lack of calibration of the blood pressure signal permitted only the analysis of SBP changes. Absolute changes in SBP would give further insight in blood pressure regulation during OM chanting. Furthermore, partial pressure of O_2_ and CO_2_ were not measured and, hence, it is not known whether these OM chanting patterns, i.e., low respiratory frequencies, lead to hypoxia or hypercapnia which also may affect cardiovascular regulation.

## Conclusion

Non-paced OM chanting significantly increases synchronisation between the rhythms of respiration, heart period and systolic blood pressure. For breathing (chanting) frequencies at approximately 0.05 Hz, two oscillations appear in heart period and systolic blood pressure during one breathing cycle: the first oscillation is induced during inspiration in the beginning of OM whereas the second oscillation is not induced by respiration. If the breathing frequency exceeds 0.065 Hz, the second oscillation in blood pressure and heart period disappears. Further systematic studies at respiratory frequencies below 0.1 Hz incorporating different inspiration to expiration ratios are needed to shed more light into the physiological control of heart period and blood pressure during consciously mediated breathing modalities.

Interestingly, most subjects choose a breathing frequency of approximately 0.05 Hz. Trained speech therapists seem to prefer a respiration frequency for OM chanting with an integer ratio (1:2) to a deeply engrained physiological rhythm, the Mayer waves (typically at approx. 0.1 Hz). In the time order, respiration always leads and is followed by the oscillations of systolic blood pressure and heart rate variability. This research shows that OM chanting has profound effects not only on respiration, but also on heart period and blood pressure. These may be applied therapeutically.

## Data Availability

The raw data supporting the conclusion of this article will be made available by the authors, without undue reservation.
